# Pre- and Post-cataract Surgery Blinking Rate as an Evaluative Indicator of Clinical Outcome

**DOI:** 10.7759/cureus.98758

**Published:** 2025-12-08

**Authors:** Aanchal Singhal, Shreya Bhadani, Soumyakanta Mohanty, Srinidhi N

**Affiliations:** 1 Ophthalmology, Kalinga Institute of Medical Sciences, Bhubaneswar, IND

**Keywords:** axial length, blink rate, dry eye, keratometry, ocular surface, phacoemulsification, schirmer, small-incision cataract surgery, tear breakup time

## Abstract

Purpose: To compare postoperative outcomes of phacoemulsification (PHACO) and small-incision cataract surgery (SICS), with emphasis on visual acuity, blink rate, ocular surface health and biometric changes.

Methods: A prospective study was conducted on 80 patients (40 PHACO, 40 SICS) with age-related cataracts. Preoperative and postoperative assessments at day one, week one, and 1.5 months included LogMAR visual acuity, blink rate, Schirmer’s test, tear breakup time (TBUT), keratometry (K1, K2), and axial length.

Results: Mean visual acuity improved markedly (LogMAR 1.57 ± 0.87 to 0.37 ± 0.25, *p*<0.001). Blink rate reduced significantly (6.63 ± 5.03 to 2.91 ± 1.84/min, *p*<0.001), reflecting better ocular surface stability. TBUT showed a modest increase (5.34 ± 2.58 to 6.38 ± 4.66 s, *p*=0.023), significant in PHACO (*p*=0.013) but not in SICS (*p*=0.114). Schirmer’s values (*p*=0.124) did not show significant overall change, though subgroup analysis revealed PHACO was associated with reduced tear secretion. Both groups showed significant increases in corneal curvature and axial length by 1.5 months.

Conclusion: Phacoemulsification provided faster and superior visual recovery but was associated with reduced tear secretion, whereas SICS preserved ocular surface comfort despite slower visual rehabilitation. Blink rate reduction and TBUT improvement emerged as simple, non-invasive indicators of postoperative ocular surface stability, supporting their incorporation into cataract surgery outcome assessment.

## Introduction

Cataract is the leading cause of avoidable blindness worldwide, accounting for nearly 51% of global cases, with India disproportionately affected, where over 60% of blindness in the elderly is attributed to cataracts [1]. Phacoemulsification (PHACO) and small-incision cataract surgery (SICS) are the two most widely practiced techniques, offering excellent visual rehabilitation. Despite consistently good outcomes in best-corrected visual acuity (BCVA), many patients report ocular surface-related symptoms such as dryness, discomfort, or visual fatigue, which can compromise postoperative satisfaction [2].

Blinking is a fundamental neuromuscular reflex that maintains ocular surface integrity by redistributing the tear film and preserving optical clarity [3]. Alterations in blink rate following cataract surgery may reflect subtle surface stress due to corneal nerve disruption, surgical inflammation, or exposure to preservative-containing medications [4,5]. 

Conventional tests such as Schirmer’s and tear breakup time (TBUT) provide useful insights into tear volume and stability but often remain impaired for weeks postoperatively [6]. Patient-reported outcomes, particularly the Ocular Surface Disease Index (OSDI), capture subjective symptoms but show variable correlation with objective parameters, especially in evaporative dry eye disease [7]. Biometric parameters such as keratometry (K1, K2) and axial length may also reflect incision-related remodelling, indirectly influencing tear distribution and surface dynamics [8].

Against this background, our study evaluated blink rate as a novel, non-invasive biomarker in the postoperative recovery phase of cataract surgery. Specifically, we compared changes in blink dynamics, ocular surface indices, and biometric parameters between PHACO and SICS to better understand their impact on visual outcomes and patient comfort.

Therefore, the present study aimed to compare postoperative outcomes between PHACO and SICS with respect to visual acuity recovery, blink rate as a functional biomarker of ocular surface stability, objective ocular surface parameters including Schirmer’s test and TBUT, and biometric changes comprising keratometry (K1, K2) and axial length. 

## Materials and methods

This longitudinal prospective observational study was conducted at the Pradyumna Bal Memorial Hospital, Kalinga Institute of Medical Sciences, Bhubaneswar, Odisha, between January 2024 and July 2025. A total of 80 patients aged 40-70 years with age-related cataracts were enrolled and randomized into two groups: Group A (n=40, phacoemulsification) and Group B (n=40, small-incision cataract surgery).

Sample size

Sample size estimation was based on Itokawa et al. [9], who reported a correlation coefficient (r = -0.392) between blink rate and TBUT, with 95% confidence and 80% power. After adjusting for an anticipated 30% attrition, the minimum required sample was 64 eyes; therefore, 80 patients were recruited to strengthen statistical validity.

Inclusion and exclusion criteria

Inclusion criteria were patients aged 40-70 years, of either gender, who were willing to undergo cataract surgery. Exclusion criteria were prior ocular surgery, neurodegenerative disease, glaucoma or ocular hypertension, patient on topical therapy, eyelid or conjunctival lesions, or use of ocular medications other than routine postoperative drops.

Preoperative evaluation

Visual acuity was assessed using both Snellen and LogMAR formats. The Snellen chart expresses vision as a fraction (e.g., 6/6, 6/60), representing the distance at which a patient can identify optotypes compared with a normal observer. The LogMAR scale provides a logarithmic representation of minimum angle of resolution, offering more precise, evenly progressive scoring suitable for statistical analysis. LogMAR values were used for statistical comparison, while Snellen values were recorded for clinical documentation. Blink rate (measured manually with a stopwatch over a three-minute period, performed by a single trained observer for all participants to prevent inter-observer variability. Assessments were carried out in a standardized environment - uniform ambient illumination, seated position, no conversation, no screen exposure or reading, and with the patient fixating straight ahead), Schirmer’s I test (without anaesthesia) and TBUT. Biometric parameters recorded were keratometry (K1, K2) and axial length (IOLMaster). Fundus examination was performed in all patients after mydriasis.

Surgical procedure

All surgeries were performed under peribulbar anesthesia by experienced cataract surgeons. The PHACO group underwent standard phacoemulsification with foldable posterior chamber intraocular lens (IOL) implantation, while the SICS group underwent manual small-incision cataract extraction with rigid posterior chamber intraocular lens implantation.

Postoperative follow-up

Follow-up was scheduled on day one, week one, and at 1.5 months. Parameters reassessed included visual acuity, blink rate, Schirmer’s test, TBUT, K1, K2, and axial length.

Statistical analysis

Data were compiled in Microsoft Excel (Microsoft, Redmond, WA, USA) and analysed using SPSS software (IBM Corp., Armonk, NY, USA). Continuous variables were expressed as mean ± standard deviation or median (Q1-Q3). Paired t-test or Wilcoxon signed-rank test was applied for within-group comparisons; Mann-Whitney U test was used for intergroup differences. Categorical data were analysed with the chi-square test. A p-value <0.05 was considered statistically significant.

## Results

Table 1 shows that the baseline characteristics of the PHACO and SICS groups were comparable, indicating appropriate randomization and absence of preoperative selection bias. Mean age and sex distribution did not differ significantly between groups. Laterality (right vs left eye) was similarly balanced. Preoperative visual acuity, blink rate, TBUT, Schirmer’s test values, keratometry (K1, K2) and axial length showed no statistically significant differences (p > 0.05), confirming that both groups started from a similar clinical profile before surgery. This comparability ensures that postoperative differences observed in the study can be attributed to the surgical technique rather than baseline variability.

**Table 1:** Baseline demographic and preoperative clinical characteristics of PHACO and SICS groups Baseline characteristics were similar between PHACO and SICS groups (p > 0.05), confirming adequate comparability prior to surgery. TBUT: tear breakup time, SICS: small-incision cataract surgery, PHACO: phacoemulsification, LogMAR = logarithm of the minimum angle of resolution.

Parameter	PHACO (n = 40)	SICS (n = 40)	p-value	Interpretation
Age (years), Mean ± SD	61.82 ± 8.45	60.47 ± 7.92	0.38	Comparable age distribution
Sex (Male/Female)	22 / 18	21 / 19	0.82	Balanced gender distribution
Affected eye (Right/Left)	19 / 21	20 / 20	0.84	No sided-eye bias
Pre-operative LogMAR visual acuity, Median (Q1–Q3)	1.30 (0.54–1.74)	2.20 (1.00–2.70)	< 0.001	PHACO group had better baseline visual acuity
Pre-operative blink rate (/min), Median (Q1–Q3)	6.00 (2.00–8.75)	5.00 (3.00–10.00)	0.44	Comparable blink dynamics at baseline
Pre-operative TBUT (seconds), Median (Q1–Q3)	4.00 (3.00–7.00)	5.00 (4.00–7.00)	0.19	Comparable tear film stability
Pre-operative Schirmer’s (mm), Median (Q1–Q3)	14.50 (5.00–20.00)	13.00 (9.00–24.00)	0.52	Comparable tear secretion

**Figure 1:**
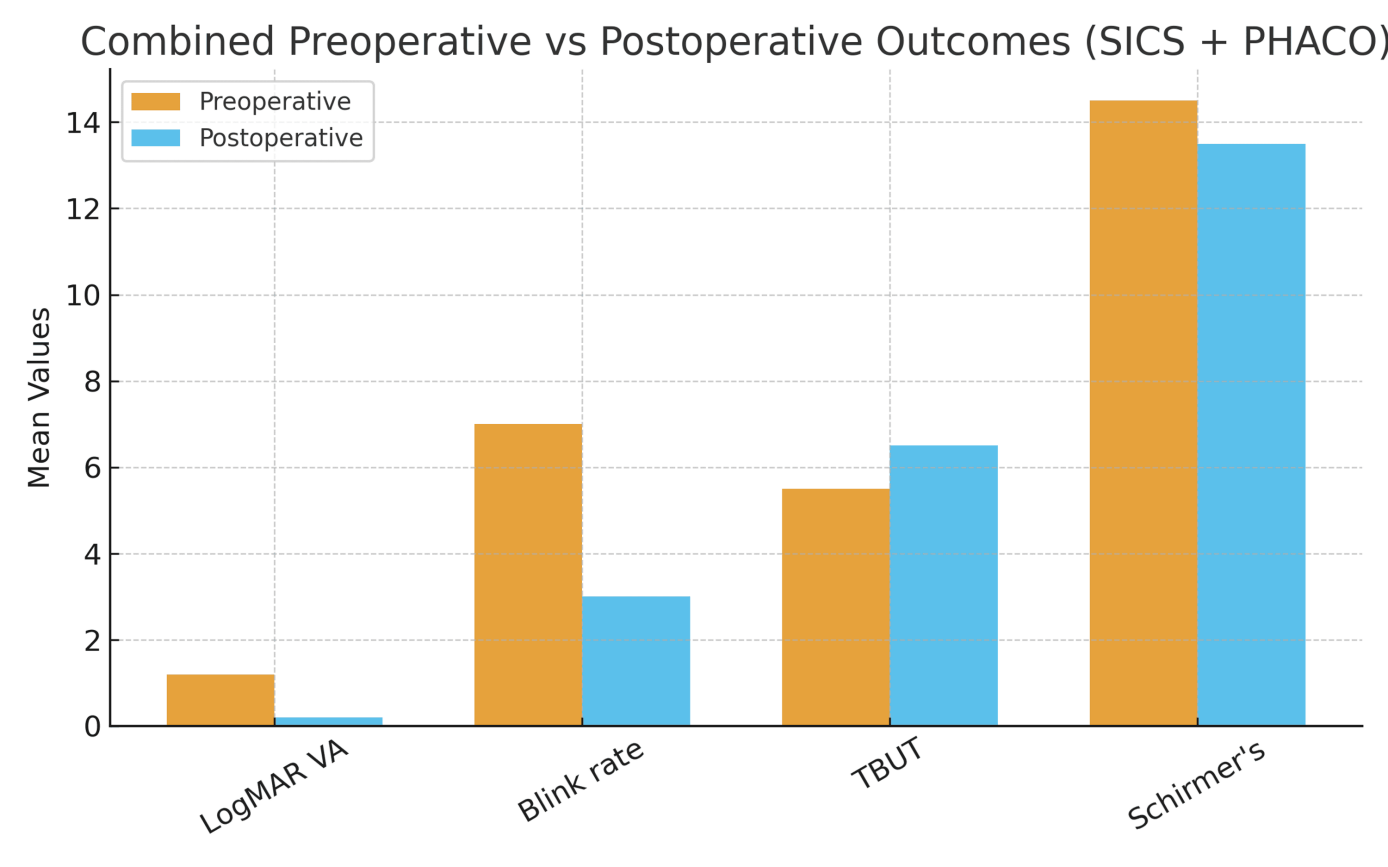
Bar chart showing changes in visual acuity, blink rate, TBUT and Schirmer’s before and after cataract surgery (combined SICS + PHACO analysis) TBUT: tear breakup time, SICS: small-incision cataract surgery, PHACO: phacoemulsification, LogMAR = logarithm of the minimum angle of resolution.

Table 2 and Figure 1 show that at 1.5 months postoperatively, visual acuity (LogMAR) demonstrated highly significant improvement across the combined cohort, encompassing both SICS and PHACO patients. The mean value improved from 1.57 ± 0.87 to 0.37 ± 0.25 (p < 0.001), reflecting restoration of functional vision and confirming the effectiveness of cataract surgery in achieving rapid and substantial rehabilitation. This level of recovery corresponds to a shift from severe baseline visual impairment to functional acuity in the range of 6/12-6/18, which directly enhances daily living and quality of life.

**Table 2:** Combined analysis of preoperative and 1.5-month postoperative outcomes (SICS + PHACO) TBUT: tear breakup time, SICS: small-incision cataract surgery, PHACO: phacoemulsification, LogMAR = logarithm of the minimum angle of resolution.

Parameter	Preoperative (Mean ± SD)	1.5-month postoperative (Mean ± SD)	p-value	Interpretation
LogMAR visual acuity	1.57 ± 0.87	0.37 ± 0.25	< 0.001	Significant visual improvement
Blink rate (/min)	6.63 ± 5.03	2.91 ± 1.84	< 0.001	Significant reduction in blink rate
TBUT (s)	5.34 ± 2.58	6.38 ± 4.66	0.023	Significant increase in tear stability
Schirmer’s (mm)	14.91 ± 9.27	13.76 ± 8.84	0.124	No significant change in tear quantity

Blinking rate decreased markedly, from 6.63 ± 5.03 to 2.91 ± 1.84 blinks/min (p < 0.001). This reduction signifies improvement in ocular surface comfort and neurosensory adaptation, as an elevated blink frequency is often a compensatory mechanism in response to ocular surface stress, tear instability, or subclinical inflammation. The normalization of blink rate therefore reflects enhanced surface stability and reduced ocular fatigue, serving as a sensitive indicator of functional recovery.

TBUT also increased significantly, from 5.34 ± 2.58 to 6.38 ± 4.66 seconds (p = 0.023). Although values remained below the normal physiological threshold (>10 seconds), the increase suggests partial restoration of tear film stability, with the ocular surface becoming less prone to premature evaporation. This improvement may be attributed to postoperative reduction in surface inflammation, improved lid-corneal interaction following clearer vision, and better distribution of tears across the corneal surface.

In contrast, Schirmer’s test values did not change significantly (14.91 ± 9.27 to 13.76 ± 8.84, p = 0.124). This stability indicates that aqueous tear production, a function of the lacrimal gland, was unaffected by cataract surgery. The absence of significant change suggests that the surgery primarily influences tear film quality rather than quantity.

**Figure 2:**
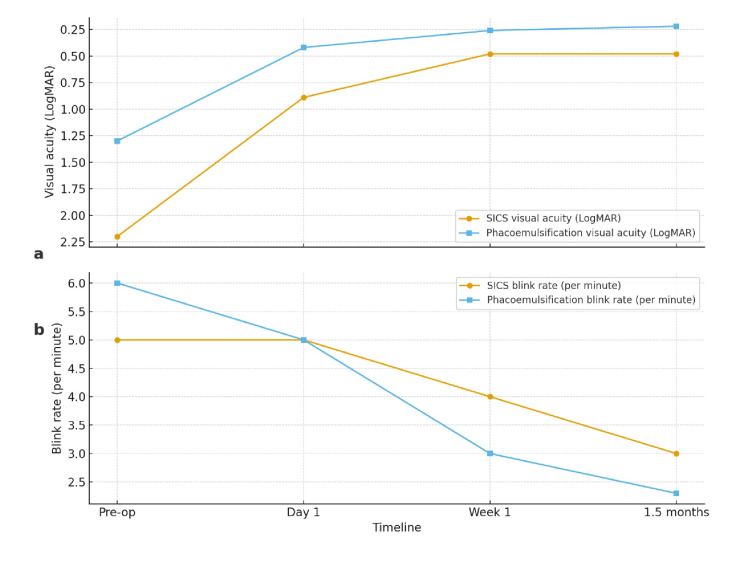
Line diagram showing timeline-based changes in visual acuity and blink rate in SICS and PHACO patients a) - Timeline based comparison of visual acuity between SICS and PHACO b) - Timeline based comparison of blinking rate between SICS and PHACO SICS: small-incision cataract surgery, PHACO: phacoemulsification, LogMAR = logarithm of the minimum angle of resolution.

Table 3 and Figure 2 show that both surgical groups demonstrated statistically significant improvement in visual acuity and reduction in blink rate over time (p < 0.001), underscoring the effectiveness of cataract surgery in both techniques. Preoperatively, the SICS group had poorer baseline vision (median LogMAR 2.20) compared with PHACO (1.30). PHACO patients achieved faster and more consistent recovery, reaching a median LogMAR of 0.22 versus 0.48 in the SICS group at 1.5 months. This highlights the superior optical quality and quicker rehabilitation associated with phacoemulsification.

**Table 3:** Timeline-based comparison of visual acuity and blink rate between SICS and PHACO SICS: small-incision cataract surgery, PHACO: phacoemulsification, LogMAR = logarithm of the minimum angle of resolution.

Parameter	Timeline	SICS (Median, Q1–Q3)	PHACO (Median, Q1–Q3)	p-value
Visual acuity (LogMAR)	Pre-op	2.20 (1.00–2.70)	1.30 (0.54–1.74)	<0.001
	Post-op Day 1	0.89 (0.39–1.00)	0.42 (0.18–1.00)	
	Post-op Week 1	0.48 (0.30–0.66)	0.26 (0.18–0.36)	
	Post-op 1.5 months	0.48 (0.30–0.60)	0.22 (0.18–0.36)	
Blink rate (/min)	Pre-op	5.00 (3.00–10.00)	6.00 (2.00–8.75)	<0.001
	Post-op Day 1	5.00 (2.00–8.00)	5.00 (2.13–7.83)	
	Post-op Week 1	4.00 (2.00–6.00)	3.00 (2.00–4.00)	
	Post-op 1.5 months	3.00 (2.00–4.00)	2.30 (2.00–3.00)	

Blinking rate declined significantly in both groups, reflecting improved ocular surface stability and neurosensory adaptation following cataract removal. In SICS, the reduction was gradual, stabilizing at 3.00 blinks/min by 1.5 months, whereas in PHACO, the decline was steeper, from 6.00 to 2.30 blinks/min, with narrower interquartile ranges suggesting more uniform recovery. Elevated baseline blink rates in PHACO may reflect greater preoperative ocular surface stress, which normalized more effectively after surgery.

Taken together, these findings demonstrate that both techniques restore vision and stabilize the ocular surface, but phacoemulsification provides superior early visual outcomes and greater normalization of blink dynamics. Clinically, this suggests PHACO may be more suitable for patients prioritizing rapid visual rehabilitation, while SICS may remain preferable in contexts where ocular surface comfort is a priority.

**Figure 3:**
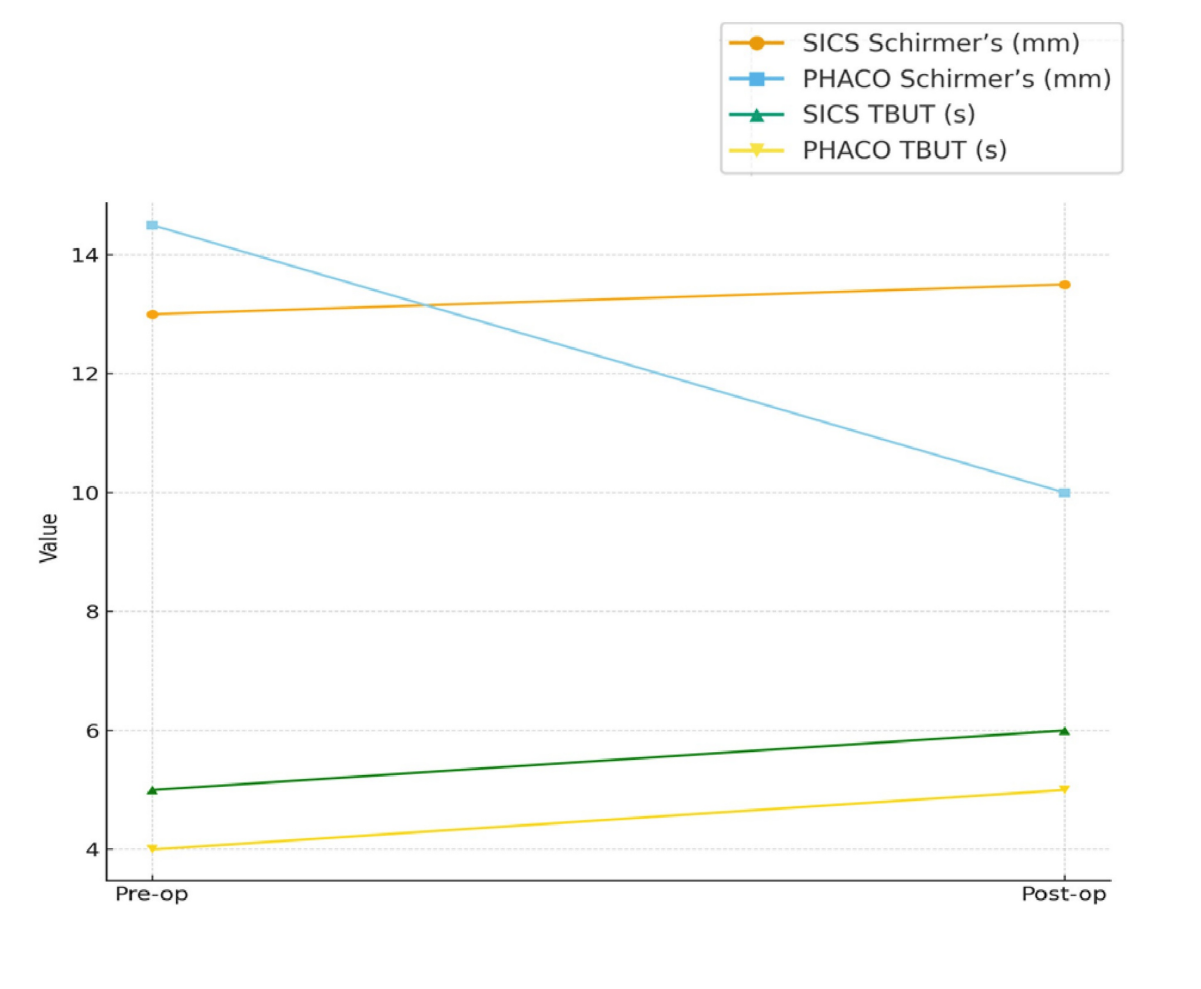
Line diagram showing timeline-based changes in ocular surface parameters in SICS and PHACO patients TBUT: tear breakup time, SICS: small-incision cataract surgery, PHACO: phacoemulsification.

Table 4 and Figure 3 show the ocular surface and biometric parameters before and after surgery for both SICS and PHACO groups. Schirmer’s test values remained relatively stable in the SICS group, indicating preserved tear secretion, while PHACO showed a significant reduction (p = 0.005), suggesting postoperative compromise of aqueous tear production. TBUT improved modestly in both groups, though statistical significance was observed only in PHACO (p = 0.013), reflecting partial recovery of tear film stability. Biometric parameters demonstrated consistent changes in both groups. Corneal curvature (K1 and K2) showed significant steepening, more marked in PHACO, attributable to incision-related remodeling and wound healing effects. Axial length increased significantly in both groups, which may reflect pseudophakic anatomical adjustments and measurement artifacts inherent to optical biometry post-IOL implantation. Collectively, these findings suggest that while both techniques alter corneal and biometric profiles, SICS better preserves tear dynamics, whereas PHACO induces greater ocular surface stress despite faster functional recovery.

**Table 4:** Ocular surface and biometric parameters before and after surgery TBUT: tear breakup time, SICS: small-incision cataract surgery, PHACO: phacoemulsification, K1: horizontal keratometry, K2: vertical keratometry, D: Diopters

Parameter	Group	Pre-op (Median, Q1–Q3)	Post-op (Median, Q1–Q3)	p-value
Schirmer’s (mm)	SICS	13.00 (9.00–24.00)	13.50 (8.00–24.50)	0.554
	PHACO	14.50 (5.00–20.00)	10.00 (5.50–16.50)	0.005
TBUT (s)	SICS	5.00 (4.00–7.00)	6.00 (4.00–8.00)	0.114
	PHACO	4.00 (3.00–7.00)	5.00 (3.25–7.00)	0.013
K1 (D)	SICS	43.98 (42.74–45.76)	44.63 (43.53–45.53)	0.029
	PHACO	43.87 (43.29–44.93)	44.65 (44.21–45.30)	<0.001
K2 (D)	SICS	44.85 (44.03–46.58)	45.66 (44.45–46.91)	<0.001
	PHACO	44.69 (44.06–45.78)	45.40 (44.88–46.60)	<0.001
Axial length (mm)	SICS	22.89 (22.05–23.32)	23.16 (22.31–24.24)	0.003
	PHACO	22.93 (22.42–23.38)	23.55 (22.99–24.05)	<0.001

## Discussion

Both phacoemulsification and SICS achieved significant improvements in visual acuity and ocular surface indices by 1.5 months, though their postoperative profiles differed. Visual rehabilitation was faster and more consistent in PHACO. A study conducted by Gogate P et al. also demonstrated that SICS patients achieved slower but stable gains, underscoring its value as a cost-effective alternative, particularly in resource-limited settings where long-term visual outcomes remain the priority [10].

Blink rate as a functional biomarker

Blink rate declined significantly in both groups, sharply in PHACO. This supports blink rate as a non-invasive biomarker of ocular surface stability, consistent with Talens-Estarelles et al., who reported reduced blink frequency after phacoemulsification [7] and Itokawa et al. who emphasized blink rate as a non-invasive marker of tear film stability [9]. Our results, therefore, suggest that blink dynamics may serve as a simple, integrative biomarker of ocular surface recovery following cataract surgery. Importantly, the decline in blink rate paralleled improvements in TBUT, supporting the physiological link between blink behavior, tear film quality, and ocular comfort.

Ocular surface outcomes

Ocular surface assessments revealed a divergence between techniques. SICS largely preserved tear secretion and stability, with Schirmer’s and TBUT values remaining stable. In contrast, PHACO patients experienced a significant reduction in Schirmer’s values despite modest TBUT improvement at 1.5 months. These results echo the observations of Park Y et al., highlighting that subjective dry eye symptoms may persist despite objective improvements, particularly following phacoemulsification [11]. For example, Sahu et al. found that after phacoemulsification, all dry eye test values (Schirmer’s, TBUT, corneal/conjunctival staining) deteriorated and subjective symptom scores rose, with many not returning to baseline by two months postoperatively [12]. Similarly, Sidaraite et al. in a comparative study of SICS and phacoemulsification noted that although objective measures improved over time, subjective complaints remained higher in the PHACO group, especially in the early post-operative period [13]. This discrepancy underscores the complexity of postoperative dry eye disease, where patient perception is influenced not only by tear metrics but also by neurosensory function, corneal nerve recovery, and possibly inflammatory or neurotrophic alterations after surgery.

Biometric changes

Biometric analysis showed mild but significant corneal steepening and axial length increases in both groups, likely reflecting incision-related remodeling and pseudophakic anatomical adjustments. Xiao et al. investigated the changes in corneal curvature and astigmatism in elderly patients with senile cataracts after phacoemulsification [14]. The study found that while the total and anterior corneal curvature significantly increased, overall astigmatism did not change significantly at the three-month mark. McQueen BR et al. explored the role of corneal topography in cataract surgery, highlighting its importance for evaluating surgical outcomes and improving preoperative planning, particularly for complex cases [15]. The authors note that while traditional keratometry was a standard tool, topography offered significant advancements by mapping the entire corneal surface, revealing details not captured by older methods. Zhao JF et al. identified significant inter-gender and inter-age differences in biometric parameters and established relationships between them, highlighting lens thickness as a key factor influencing anterior chamber depth and suggesting that axial length is a primary factor affecting refractive prediction accuracy in cataract surgery [16]. Goto S et al. further demonstrated that while early keratometric and axial variations occur, long-term measurements remain stable and reliable for surgical planning [17]. Importantly, in our study these changes stabilized by 1.5 months, with no long-term intergroup differences, suggesting comparable anatomic stability of both techniques.

Clinical implications

The differing postoperative trajectories between PHACO and SICS provide valuable insights into patient counseling and surgical planning. While PHACO ensures faster recovery and clearer vision in the early postoperative period, it also appears to predispose patients to a higher dry eye burden due to decreased tear production, likely related to greater corneal nerve disruption and cumulative energy delivery. In contrast, SICS, though slower in visual rehabilitation, preserves ocular surface homeostasis more effectively, making it particularly suitable for patients at high risk of postoperative dry eye (e.g., elderly, pre-existing meibomian gland dysfunction, or systemic autoimmune disease). Blink dynamics, as demonstrated in this study, may complement conventional tear tests by offering a quick, patient-friendly assessment of ocular comfort and tear stability. Incorporating blink rate into postoperative protocols could enable clinicians to identify at-risk patients earlier, guide the use of preservative-free lubricants, and tailor postoperative medication regimens to mitigate ocular surface stress. Beyond routine care, these findings highlight the potential for blink rate monitoring to be integrated into digital health platforms or automated video-based systems, expanding its utility as a cost-effective biomarker in both clinical and community screening contexts.

Limitations

This study has several limitations. It was conducted at a single center with a modest sample size and a relatively short follow-up period (1.5 months), which may restrict generalizability. The allocation of PHACO versus SICS was based on cataract grade rather than full randomization, introducing potential selection bias. Blink rate was measured manually without complete environmental standardization, and masking of evaluators was not performed, creating a risk of observer bias. Intraoperative variables such as PHACO energy, incision size, and postoperative drug regimens were not controlled or analyzed separately and may have influenced ocular surface outcomes. Future multicenter studies with longer follow-up and automated blink-tracking systems are warranted to validate these findings.

## Conclusions

Both SICS and PHACO provide substantial visual rehabilitation. PHACO ensures faster recovery but is associated with postoperative dry eye, while SICS preserves tear function and comfort despite slower rehabilitation. Blink rate reduction emerged as a novel, non-invasive biomarker correlating with ocular surface stability and patient comfort, supporting its inclusion in routine postoperative monitoring. In addition, integrating blink dynamics with conventional tear metrics may enhance individualized postoperative care and allow earlier identification of patients at risk for ocular surface morbidity. Future multicentre studies with larger cohorts, longer follow-up, and automated blink tracking technologies are warranted to validate blink dynamics as a reliable clinical tool in cataract surgery outcomes.
